# A systemic and molecular study of subcellular localization of SARS-CoV-2 proteins

**DOI:** 10.1038/s41392-020-00372-8

**Published:** 2020-11-17

**Authors:** Jing Zhang, Ruth Cruz-cosme, Meng-Wei Zhuang, Dongxiao Liu, Yuan Liu, Shaolei Teng, Pei-Hui Wang, Qiyi Tang

**Affiliations:** 1 Advanced Medical Research Institute, Cheeloo College of Medicine, Shandong University, Jinan, Shandong 250012 China; 2 Howard University College of Medicine, 520 W Street NW, Washington, DC 20059 USA; 3 Weill Institute for Cell and Molecular Biology, Cornell University, Ithaca, NY USA; 4 Department of Biology, Howard University, 415 College St. NW, Washington, DC 20059 USA

**Keywords:** Microbiology, Cell biology, Vaccines

**Dear Editor**,

The current pandemic of beta-coronavirus (SARS-CoV-2) has exerted devastating influence on almost all countries, resulting in the disease named COVID-19.^1^ Coronavirus possesses the largest RNA genome among all the RNA viruses. Its genome encodes about 29 proteins (Supplementary Fig. S1). The subcellular distributions of the viral proteins have yet been reported for SARS-CoV-2. It is important to investigate the viral proteins’ locations in cells because the subcellular distribution information not only helps us in understanding how viruses interact with the host cells but also provides clues in fighting against the viral infection. Therefore, we cloned all the genes of SARS-CoV-2 into vectors for expression in mammalian cells and used immunofluorescent assay (IFA) to examine the viral proteins’ subcellular location. Except for the NSP11 that is only 14 aa long, we expressed all other 28 viral proteins in HEp-2 or Caco-2 cells and found a diversity of protein distribution in cells, suggesting a complicated interaction of SARS-CoV-2 with host cells to achieve a successful infection.

In a systemic attempt of revealing the subcellular locations of SARS-CoV-2 proteins, we transfected each plasmid into HEp-2 cells for 20 h, then the cells were fixed for IFA using anti-FLAG antibody to show the viral protein and anti-CoxIV to show the mitochondria or anti-Giantin to show the Golgi apparatus.

As can be seen in the Supplementary Fig. S2, the viral proteins are either cytoplasmic (NSP2, NSP3C, NSP4, NSP8, Spike, M, N, ORF3a, ORF3b, ORF6, ORF7a, ORF7b, ORF8, ORF9b and ORF10) or both nuclear and cytoplasmic (NSP1, NSP3N, NSP5, NSP6, NSP7, NSP9, NSP10, NSP12, NSP13, NSP14, NSP15, NSP16, E and ORF9a). Although no viral proteins were detected in mitochondria, M protein colocalizes with Giantin, which is consistent to that of SARS-CoV-1. Whether other proteins are related to Golgi apparatus or other cellular organelles needs to be further investigated. Interestingly, some proteins showed punctate staining in the IFA experiments: NSP1, NSP5, NSP9, NSP12, NSP13, NSP14, NSP15, ORF3a and M. The relationships of these proteins with subcellular organelles are further explored in this study.

Results from the Supplementary Fig. S2 showed that some proteins are cytoplasmic punctate proteins. We wondered if they are in any cellular organelles. First, we examined their locations with Golgi apparatus. The HEp-2 or Caco-2 cells were fixed at the 24 h post-transfection and stained with anti-FLAG in green and anti-Giantin to visualize the Golgi apparatus in red. Consequently, we detected that four SARS-CoV-2 proteins are related to Golgi apparatus: NSP15, M, ORF6 and ORF7a. As shown in Fig. 1a (left) and the Supplementary Fig. S3a, viral proteins M, ORF7a and NSP15 colocalize with Golgi apparatus, and ORF6 partially colocalizes with Golgi apparatus. Except that M-Golgi apparatus relationship has been previously reported,^2^ other proteins’ relationships with Golgi apparatus are the first reported by this study.

**Fig. 1:**
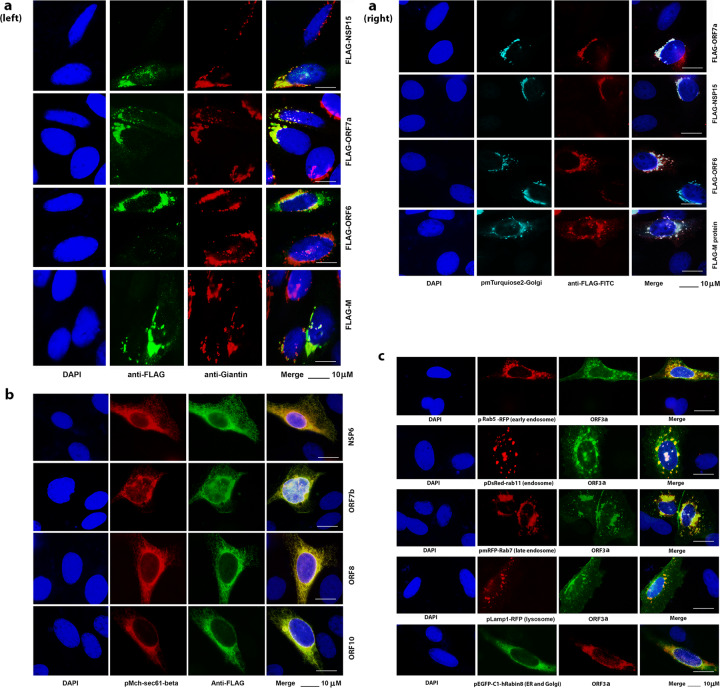
Subcellular locations of SARS-CoV-2 proteins. IFA was performed at 24 h after transfection of the plasmid expressing the viral protein into HEp-2 cells. **a** NSP15, M, ORF6, and ORF7a, are associated with Golgi apparatus. **b** NSP6, ORF7b, ORF8 and ORF10, are related to ER. **c** ORF3a, is related to endosome and lysosome. Bar = 10 μm

To ensure the specificities of the IFA results, we employed a co-transfection system using a Golgi apparatus protein expression plasmid in which the N-terminus (1–61 aa) of the Beta-1,4-galactosyltransferase 1 was fused with a cyan fluorescent protein variant, mTurquoise2.^3^ In this system, we only need to stain the viral proteins with anti-FLAG antibody. As shown in Fig. 1a (right) and the Supplementary Fig. S3b, the co-transfected cells were fixed for IFA and the viral proteins were immuno-stained in red fluorescence. After merging different colors, the results showed ORF6, ORF7a and NSP15, like M protein, colocalized with Golgi apparatus. Therefore, we identified four SARS-CoV-2 proteins (M, ORF6, ORF7a and NSP15) that are related to Golgi apparatus.

Like other positive-stranded RNA viruses, SARS-CoV-2 RNA is transported to endoplasmic reticulum (ER) after viral entry. ER is the major cellular organelle that viruses need to usurp because it is a factory for production of viral proteins. Most proteins of SARS-CoV-2 were seen in cytoplasm as shown in the Supplementary Fig. S2, so we asked whether they colocalize with ER. To that end, we cotransfected several viral protein-expressing plasmids (NSP6, ORF7b, ORF8 and ORF10) together with pmcCh-sec61-beta (ER and the ER-Golgi apparatus intermediate compartment). ER is in red fluorescence because it is tagged with mCherry. The viral proteins (NSP6, ORF7b, ORF8 and ORF10) were shown in green fluorescence by anti-FLAG. Although SARS-CoV-2 proteins are all generated in ER, IFA found only NSP6, ORF7b, ORF8 and ORF10 colocalized with ER as shown in Fig. 1b and the Supplementary Fig. S3c. The yellow color in the merged pictures was caused by the colocalization between the viral proteins and ER protein: sec61 beta. ORF7b is a 43 aa protein, ORF8 has only 121 aa and ORF10 contains 38 aa. Although they are small proteins, their functions might be important for viral replication and need to be further investigated.

Endosome is a cellular organelle with a membrane in eukaryotic cells and undergoes a maturation from early endosome to late endosome depending on acidification. The late endosome then fuses with the lysosome to degrade the molecule by lysosomal hydrolytic enzymes. Here we used the plasmids expressing the proteins standing for early endosome (Rab5), endosome (Rab11), late endosome (Rab7), and lysosome (Lamp1),^4^ which were cotransfected with SARS-CoV-2 protein-expressing plasmids. We identified ORF3a to be the viral protein that is associated with the formation of endosome and lysosome (Fig. 1c and the Supplementary Fig. S3d). Our IFA results showed that only ORF3a is associated with endosome and lysosome. To confirm the specificity of our IFA assay using the co-transfection system, we also co-transfected ORF3a-expressing plasmid with an ER & Golgi apparatus intermediate protein, Rabin8 that is tagged with GFP. No significant colocalization was detected between ORF3a and Rabin8. Therefore, ORF3a protein is related to the endocytosis-related biological activities. Interestingly, for the first time, we found that the N protein colocalizes with lipid droplet (LD) that was visualized by BODIPY 500/510 in the Caco-2 cells (Supplementary Fig. S4).

Interestingly, some SARS-CoV-2 proteins are detected in nucleus such as NSP1, NSP5, NSP9 and NSP13 as shown in the Supplementary Fig. S2. For these nuclear proteins, we decided to know if they interact with any nuclear structures such as SC (splicing compartment) that is important for gene splicing. As shown in the Supplementary Fig. S5, we didn’t detect any relationship between NSP1 and SC35. Both NSP5 and NSP9 distribute diffusely in the nucleus, but in the strongly stained spots of NSP5 or NSP9, SC35 appears to colocalize with the viral proteins. Interestingly, NSP13 exists in the nuclei of HEp-2 cells as round “dots” (shown by white arrows) that exactly colocalize with SC35 (Supplementary Fig. S5). This phenomenon was also found for Zika virus that NS5 to interact with SC35.^5^ A similar experiment was performed in Caco-2 cells for NSP13. We found the same results that NSP13 colocalizes with SC35 in the nuclei (Supplementary Fig. S6).

In summary, we molecularly cloned all the genes of SARS-CoV-2 and applied a systemic IFA to characterize the subcellular distribution of the viral proteins. Our results provide the field with new insight into the biological functions of SARS-CoV-2 proteins because the localization of the protein to the site of a cell implies that the protein might play its biological function in the subcellular location. However, a detailed study should be conducted in the context of SARS-CoV-2 infection in cells because viral proteins from transfection may behave differently than that from viral infection.

## Supplementary information

A Systemic and Molecular Study of Subcellular Localization of SARS-CoV-2 Proteins

## References

[1] Teng S, Tang Q (2020). ACE2 enhance viral infection or viral infection aggravate the underlying diseases. Comput. Struct. Biotechnol. J..

[2] Ujike M, Taguchi F (2015). Incorporation of spike and membrane glycoproteins into coronavirus virions. Viruses.

[3] Goedhart J (2012). Structure-guided evolution of cyan fluorescent proteins towards a quantum yield of 93%. Nat. Commun..

[4] Stenmark H (2009). Rab GTPases as coordinators of vesicle traffic. Nat. Rev. Mol. Cell Biol..

[5] Hou W (2017). Molecular cloning and characterization of the genes encoding the proteins of Zika virus. Gene.

