# Correction: A systemic and molecular study of subcellular localization of SARS-CoV-2 proteins

**DOI:** 10.1038/s41392-021-00564-w

**Published:** 2021-05-13

**Authors:** Jing Zhang, Ruth Cruz-cosme, Meng-Wei Zhuang, Dongxiao Liu, Yuan Liu, Shaolei Teng, Pei-Hui Wang, Qiyi Tang

**Affiliations:** 1 Key Laboratory for Experimental Teratology of Ministry of Education and Advanced Medical Research Institute, Cheeloo College of Medicine, Shandong University, Jinan, Shandong 250012 China; 2 Howard University College of Medicine, 520 W Street NW, Washington, DC 20059 USA; 3 Weill Institute for Cell and Molecular Biology, Cornell University, Ithaca, NY USA; 4 Department of Biology, Howard University, 415 College St. NW, Washington, DC 20059 USA

**Keywords:** Microbiology, Cell biology, Vaccines

Correction to: *Signal Transduction and Targeted Therapy* 10.1038/s41392-020-00372-8, published online 17 November 2020

Since the acceptance and publication of this article^1^, the authors noted that some funding was included by mistake. Therefore, we would like to change the ACKNOWLEDGEMENTS to.

This study was supported by National Institute on Minority Health and Health Disparities of the National Institutes of Health under Award Number G12MD007597, and by grants from COVID-19 emergency tackling research project of Shandong University (Grant No. 2020XGB03 to P.-H.W.). We thank Translational Medicine Core Facility of Shandong University for consultation and instrument availability that supported this work.
