# Supplementary material for: A systemic and molecular study of subcellular localization of SARS-CoV-2 proteins
Source: Signal Transduct Target Ther. 2020 Nov 17;5:269. doi: 10.1038/s41392-020-00372-8 (PMC7670843; doi:10.1038/s41392-020-00372-8)
Supplement: Supplementary file 1 — A Systemic and Molecular Study of Subcellular Localization of SARS-CoV-2 Proteins [file 41392_2020_372_MOESM1_ESM.pdf]

Supplementary Materials for

A Systemic and Molecular Study of Subcellular Localization of SARS-CoV-2 Proteins

Jing Zhang[1#], Ruth Cruz-cosme[2#], Meng-Wei Zhuang[1], Dongxiao Liu[2], Yuan Liu[4], Shaolei Teng[3], Pei-Hui Wang[1]* and Qiyi Tang[2]*

Correspondence to: (qiyi.tang@howard.edu) and pei-hui.wang@sdu.edu.cn or pei-hui.wang@connect.hku.hk

[1] Advanced Medical Research Institute, Cheeloo College of Medicine, Shandong University, Jinan, Shandong 250012, China.
[2] Howard University College of Medicine, 520 W Street NW, Washington, DC 20059. Tel: 202 806 3915;
[3] Department of Biology, Howard University, 415 College St. NW, Washington, DC 20059
[4] Weill Institute for Cell and Molecular Biology, Cornell University, Ithaca, NY, USA

# Principal co-authors with equal contributions

This PDF file includes:

Materials and Methods
Supplementary Text
Figures. S1 to S6
Tables S1

Materials and Methods

Molecular cloning. The sequence of severe acute respiratory syndrome coronavirus 2 (SARS-CoV-2, GenBank: NC_045512.2) isolate, Wuhan-Hu-1, was used in this study for DNA synthesis of each gene (General Bio, China). Among them, the DNA sequences of non-structure protein (NSP)3C, NSP10, open reading frame (ORF)3b, and ORF10 were codon optimized to get a high expression level in human cells. The viral genes were firstly cloned into pcDNA6B-FLAG vector using the Seamless Cloning Kit (Beyotime Biotechnology, China) or standard molecular cloning methods. To obtain a high expression level, these viral genes were then subcloned into pCAG-FLAG. The information about all SARS-CoV-2 genes was listed in Table S1. We designed the cloning strategies according to the gene structure published in https://viralzone.expasy.org/8996 with slight modification: 1) ORF3a and ORF3b are the genes after S gene; 2) following the ORF8 are the ORF9a (was ORF9b), ORF9b (was ORF14) and the last one, ORF10 (Supplementary Fig. S1). The exact positions of the genes in the viral genome and their cloning primers are listed in Table S1. Among the 28 cloned genes of SARS-CoV-2 (NSP-1 through -16 except NSP11, S, M, N, E, ORF3a, ORF3b, ORF6, ORF7a, ORF7b, ORF8, ORF9a, ORF9b and ORF10), their expressions in HEp-2 cells are all detected. A full length NSP3 cloning and expression has not been successful, so we cloned NSP3N and NSP3C both of which can be expressed. The DNA sequences are all correct and the fusion with the FLAG in frame.

Cell lines and Tissue culture. HEp-2 cells (ATCC®CCL-23-37™) were purchased from ATCC and Caco-2 (ATCC®HTB-37™) was a gift from Dr. Wenzhe Ho (Temple University, Phialdelphia). The cells were maintained in Dulbecco's modified Eagle's medium (DMEM) supplemented with 10% fetal calf serum (FCS) and penicillin (100 IU/ml)-streptomycin (100 μg/ml) and amphotericin B (2.5 μg/ml).

Plasmids and transfection. To show a protein's location in the HEp-2 cells, the plasmid constructed from "molecular cloning" is co-transfected with one of the following plasmids that expresses a cell marker. pmTurquiose2-Golgi apparatus (Beta-1,4-galactosyltransferase 1 1-61 aa) in red is to show the Golgi apparatus (Addgene cat 36205); pMch-sec61-beta shows ER and the ER-Golgi apparatus intermediate compartment (Addgene cat 49155); pLamp1-RFP represents lysosome (Addgene cat 1817); pDsRed-rab11 visualizes endosome; pmRFP-Rab7 shows late endosome; pmRFP-Rab5 shows early endosome; and pEGFP-C1-hRabin8 shows the intermediate cisternae between ER and Golgi apparatus. These plasmids are purchased from Addgene (http://www.addgene.org). Transfection reagent, Lipofectamine 3000, was purchased from Invitrogen and used according to the manufacturer's protocol.

Antibodies. Anti-Giantin (ab80864) for visualizing Golgi apparatus, and anti-CoxIV (ab16056) for showing mitochondria were purchased from Abcam (Cambridge, MA). Antinti-Tubulin (4G1, sc-58666) was purchased from Santa Cruz Biotechnology (Santa Cruz, CA). Anti-FLAG antibody (monoclonal), M2, and the anti-SC35 antibody (S4045) were purchased from Sigma.

Immunofluorescent assay (IFA). Immunostaining was performed on cells grown on coverslips after fixation with 1% paraformaldehyde (10 min at room temperature) and permeabilization in 0.2% Triton (20 min on ice) by sequential incubation with primary and Texas red (TR)-labeled secondary antibodies (Vector Laboratories, Burlingame, Calif.) for 30 min each (all solutions in PBS). Finally, cells were equilibrated in PBS, stained for DNA with Hoechst 33258 (0.5 μg/ml), and mounted in Fluoromount G (Fisher Scientific, Newark, Del.). To visualize lipid droplet (LD), the fixed cells were incubated with BODIPY 500/510 (Life Technology Corp. cat# B3824) at a final concentration of 1 μg/ml for 30 min at 37°C.

Confocal microscopy. Cells were examined with a Leica TCS SPII confocal laser scanning system. Two or three channels were recorded simultaneously and/or sequentially and controlled for possible breakthrough between the fluorescein isothiocyanate and Texas Red signals and between the blue and red channels.

Supplementary Text

The major contribution of this study to the field of COVID-19 is that we have systemically investigated the subcellular distribution of SRAS-CoV-2 proteins. To our knowledge, it is for the first time to clone all the genes of SARS-CoV-2 except NSP11 that is only 14 aa long. In addition, full length NSP3 was cloned but failed to be expressed, so we separated NSP3 into NSP3N and NSP3C. Our overarching findings include that most SARS-CoV-2 proteins are cytoplasmic, and some are both cytoplasmic and nuclear proteins, that 4 proteins in total are detected in Golgi apparatus, that ORF3a colocalizes with endosome and lysosome, and that NSP6, ORF7b, ORF8 and ORF10 colocalize with ER. Although M protein in Golgi apparatus was previously reported for SARS-CoV-1, it is interesting that M protein is not the only one associated with Golgi apparatus, suggesting that M protein might not be sufficient to ensure the maturation of E and S proteins, some non-structural proteins such as NSP15, ORF6 and ORF7a might be needed for viral maturation. The novel information might implicate biological functions for the viral proteins and help developing new anti-viral strategies against COVID-19. ORF3a colocalizes with early endosome protein Rab5, recycling endosome protein Rab11, late early some protein Rab7 and lysosome protein Lamp1, suggesting the ORF3a protein participated in the endocytic procedure that finally leads the fusion of endosome and lysosome. Whether or not the ORF3a involves in the cellular self-clearance of the toxic materials so that the infected SARS-CoV-2 replicate better remains to be investigated.

Fig S1

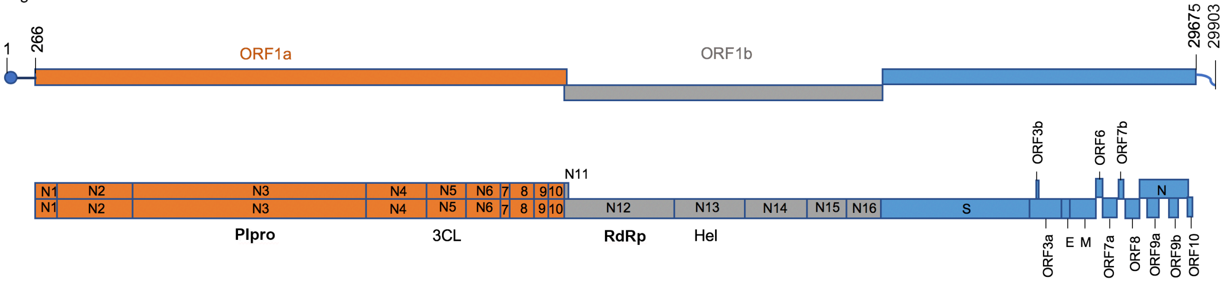

Abbreviations
N: NSP, **Plpro:** Papain-like protease, 3CL: chymotrypsin-like protease, **RdRp:** RNA-dependent RNA polymerase, Hel: Helicase, S: spike, O: ORF, E: Envelop, M: membrane, N: nucleocapsid

Fig. S1. Diagram of SARS-CoV-2 genome and the names of viral genes. The genome of Wuhan-Hu-1 (NC_045512.2) has 29,903 nucleotides and encodes 12 putative open reading frames responsible for about 29 proteins. The first gene, NSP1 starts from 266, and the last gene, ORF10 ends at 29674. The nucleic acids from 1-265 is modified by methylation to form the RNA cap, and from 29675 to 29903 is the 3' UTR.

Fig. S2

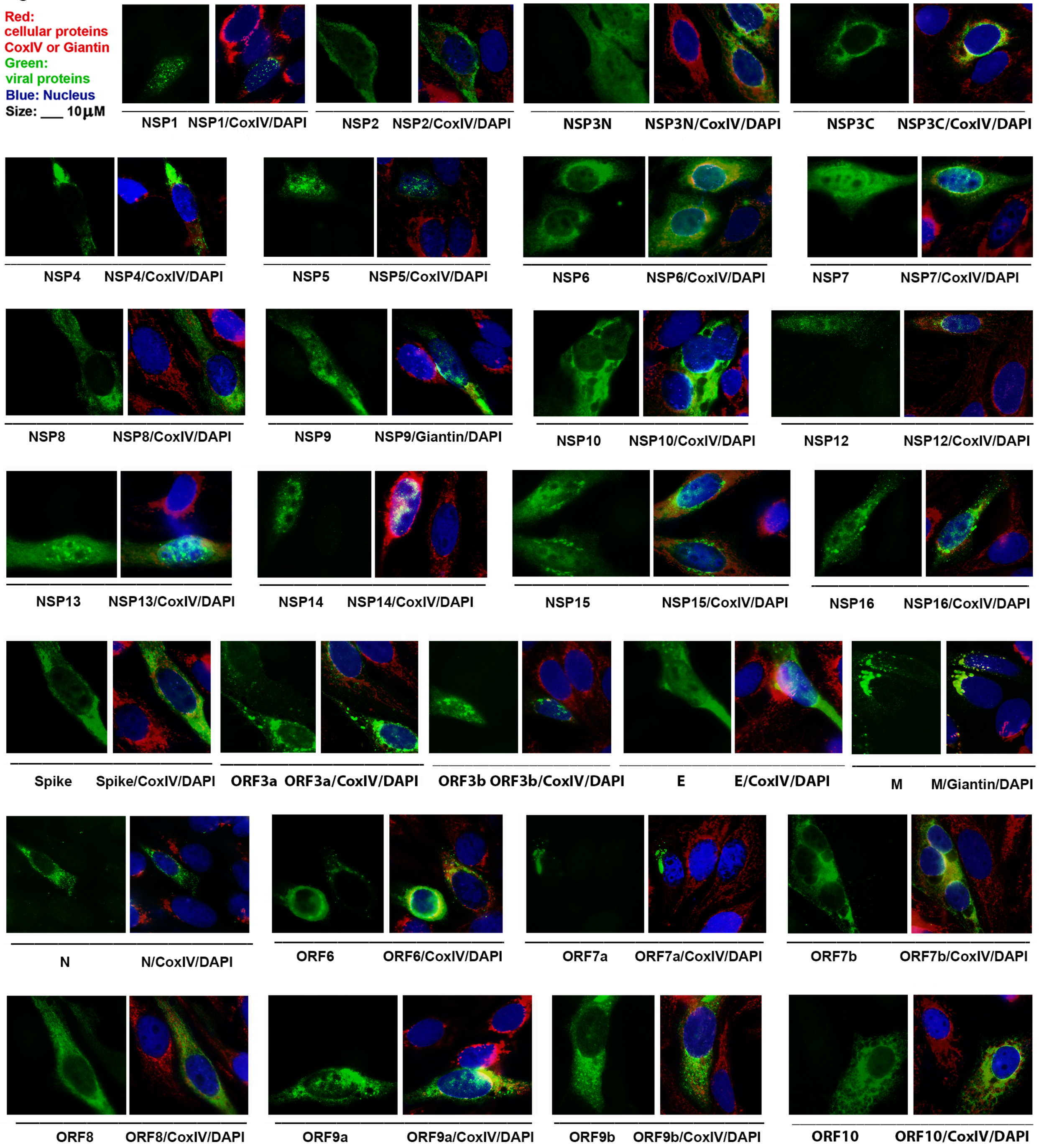

Fig. S2. SARS-CoV-2 proteins expressed in human cells. The viral gene fragments (names of the genes are as listed in the Supplementary Fig. 1 and Table 1) were synthesized with a FLAG tag at their 3' end. Each plasmid was transfected to HEp-2 cells for 20 hours. Immunohistochemistry (IHC) was performed to examine viral protein using anti-FLAG antibody in green (FITC-conjugated 2ndary antibody) and cellular proteins using anti-CoxIV or anti-Giantin antibody in red (Texas red-conjugated 2ndary). The merged pictures include DAPI to show nucleus in blue. Scale bar: 10 μm.

Fig. S3

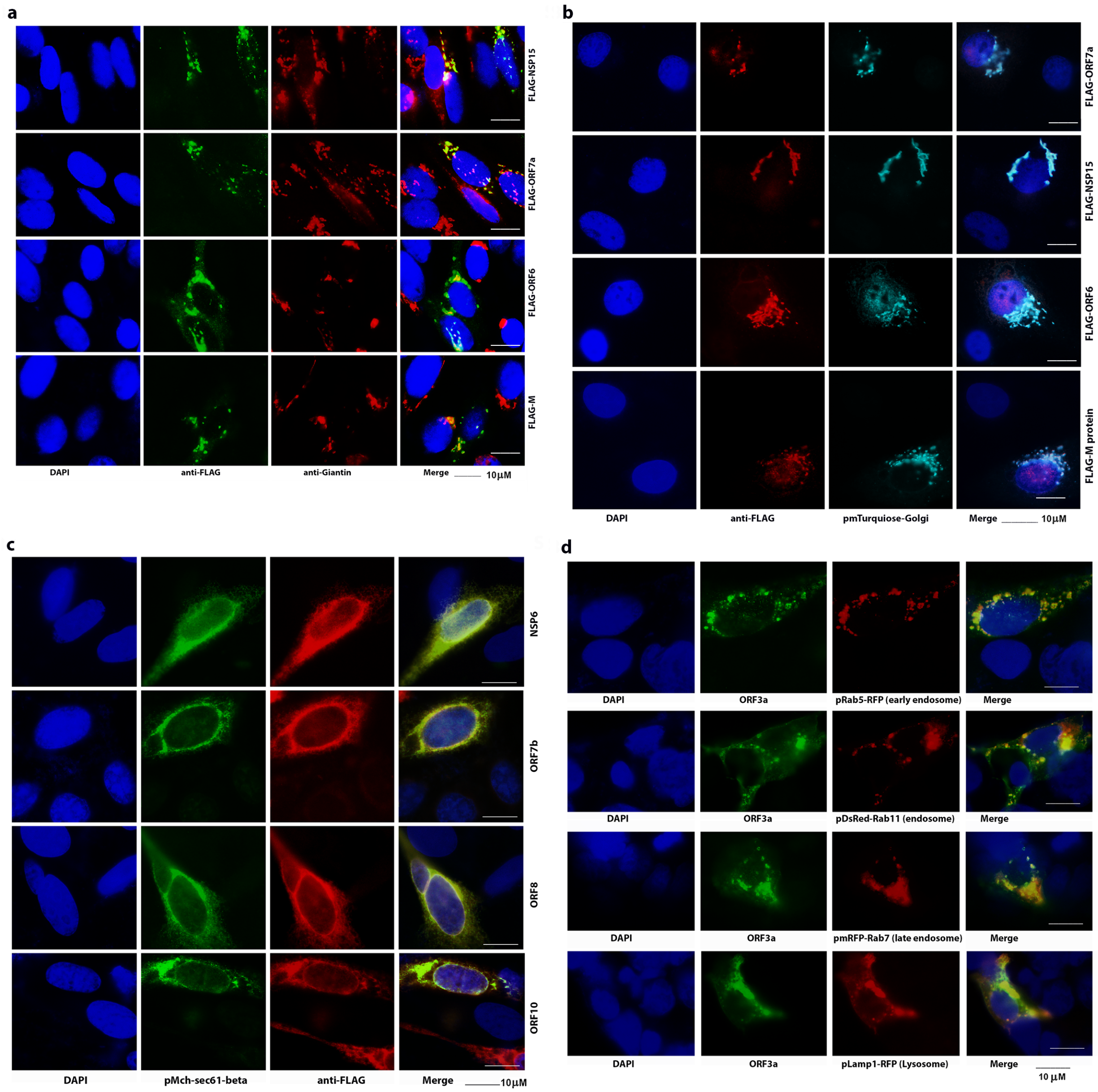

Fig. S3. Subcellular locations of SARS-CoV-2 proteins in Caco-2 cells. IFA was performed at 24 hours after transfection of the plasmid expressing the viral protein to Caco-2 cells. **a and b.** NSP15, M, ORF6, and ORF7a, are associated with Golgi apparatus. **c.** NSP6, ORF7b, ORF8 and ORF10, are related to ER. **d.** ORF3a, is related to endosome and lysosome. Bar: 10 μm. Bar: 10 μm.

Fig. S4

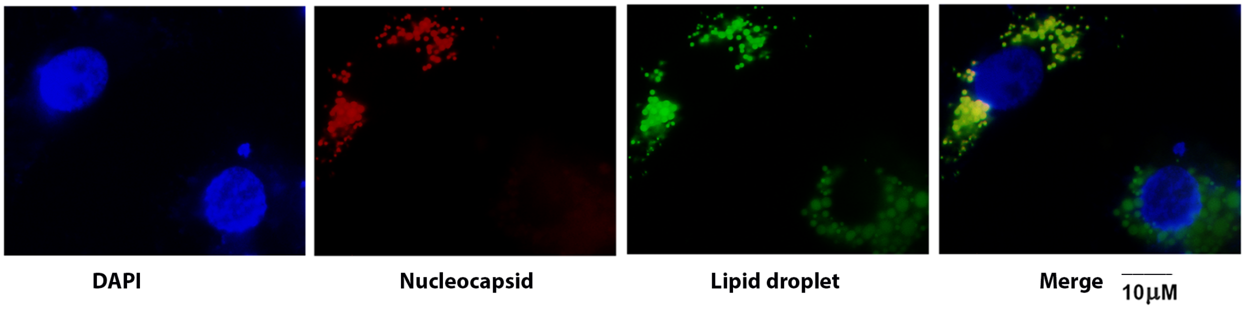

Fig. S4. SARS-CoV-2 nucleocapsid (N) protein colocalizes with lipid droplet (LD) in Caco-2 cells. The N protein-expressing plasmid was transfected to Caco-2 cells for 20 hours and the cells were incubated with 1:1000 diluted BODIPY 500/510 (Life Technology Corp. cat# B3824) for 30 min at 37°C to show LD in green. The cells were then fixed and stained with anti-FLAG antibody to show N protein in red. Bar: 10 μm.

Fig. S5

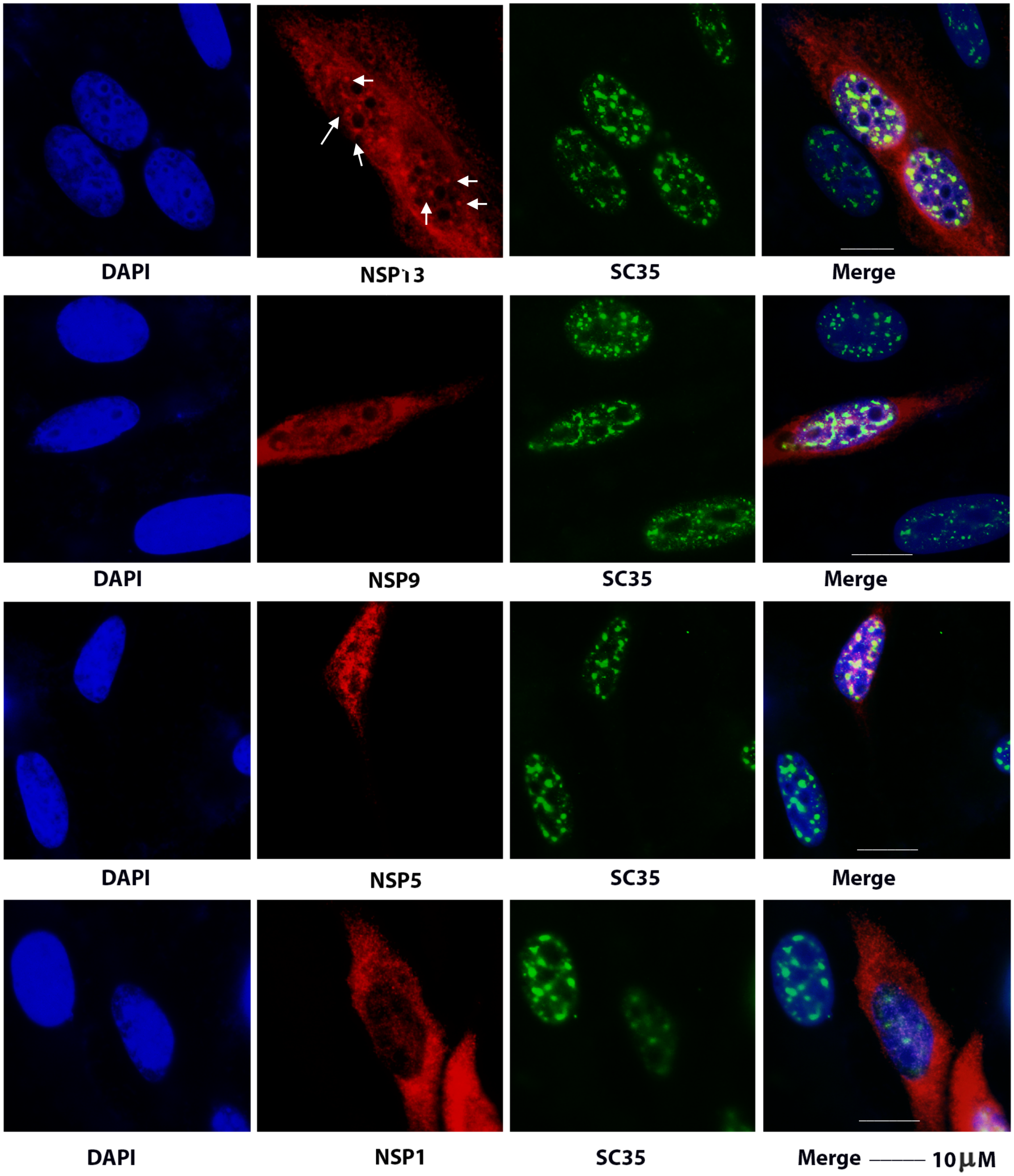

Fig. S5. SARS-CoV-2 proteins in nucleus. The plasmid expressing the viral protein as indicated was transfected to HEp-2 cells for 24 hours. IFA was performed using anti-FLAG antibody to show viral protein in red and anti-SC35 antibody to show SC in green. Bar: 10 µm. White arrows: to show the NSP13 dots in nuclei.

Fig. S6

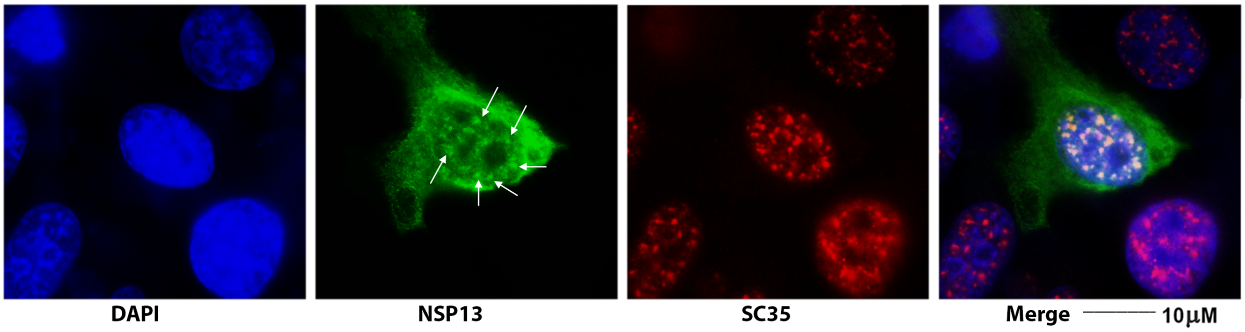

Fig. S6. SARS-CoV-2 protein, NSP13 colocalizes with SC35 in Caco-2 cells. The plasmid expressing the NSP13 protein as indicated was transfected to Caco-2 cells for 24 hours. IFA was performed using anti-FLAG antibody to show NSP13 in green and anti-SC35 antibody to show SC in red. Bar: 10 μm. White arrows: to show the NSP13 dots in nuclei.

SARS-CoV-2 Protein	Clone nt	Primers (5'-3') for cloning into pcDNA6B-FLAG
**Non-structural proteins**		
NSP1	266-805	FP: GCACAGTGGCGGCCGCTCGAGGCCACCATGGAGAGCCTTGTCCCTGG
		RP: GTCATCCTTGTAATCTCTAGACCCTCCGTTAAGCTCACGC
NSP2	806-2719	FP: GCACAGTGGCGGCCGCTCGAGGCCACCATGGCATACACTCGCTATGTCGATAAC
		RP: GTCATCCTTGTAATCTCTAGAACCGCCTTTGAGTGTGAAGG
NSP3N	2720-4504	FP: GCACAGTGGCGGCCGCTCGAGGCCACCATGGCACCAACAAAGGTTACTTTT
		RP: GTCATCCTTGTAATCTCTAGACTCTTGTATTTTAATACCCTTATATTTAC
NSP3C(opt)	4955-8554	FP: CTTGGTACCGAGCTCGGATCCGCCACCATGGAGGTGAGGACCATCAAG
		RP: GTCATCCTTGTAATCTCTAGAGCCGCCCTTCAGGGCGAT
NSP4	8555-10054	FP: GCACAGTGGCGGCCGCTCGAGGCCACCATGAAAATTGTTAATAATTGGTTGAAGC
		RP: GTCATCCTTGTAATCTCTAGACTGCAAAACAGCTGAGGTGATA
NSP5	10055-10972	FP: GCACAGTGGCGGCCGCTCGAGGCCACCATGAGTGGTTTTAGAAAAATGGCATTC
		RP: GTCATCCTTGTAATCTCTAGATTGGAAAGTAACACCTGAGCATT
NSP6	10973-11842	FP: GCACAGTGGCGGCCGCTCGAGGCCACCATGAGTGCAGTGAAAAGAACAATCAAG
		RP: GTCATCCTTGTAATCTCTAGACTGTACAGTGGCTACTTTGATACAAG
NSP7	11843-12088	FP: GCACAGTGGCGGCCGCTCGAGGCCACCATGTCTAAAATGTCAGATGTAAAGTGCAC
		RP: GTCATCCTTGTAATCTCTAGATAAGGTTGCCCTGTTGTCCA
NSP8	12089-12685	FP: GCACAGTGGCGGCCGCTCGAGGCCACCATGCAAGCTATAGCCTCAGAGTTTAGTTC
		RP: GTCATCCTTGTAATCTCTAGACTGTAATTTGACAGCAGAATTGG
NSP9	12686-13024	FP: GCACAGTGGCGGCCGCTCGAGGCCACCATGAATAATGAGCTTAGTCCTGTTGCAC
		RP: GTCATCCTTGTAATCTCTAGATTGTAGACGTACTGTGGCAGCTAA
NSP10(opt)	13025-13441	FP: CTTGGTACCGAGCTCGGATCCGCCACCATGGCCGGAAACGCCACA
		RP: GTCATCCTTGTAATCTCTAGACTGCAGCATGGGCTCCCTC
NSP11	13442-13483	FP: TCGAGGCCACCATGTCAGCTGATGCACAATCGTTTTTAAACGGGTTTGCGGTGTAAT
		RP: CTAGATTACACCGCAAACCCGTTTAAAAACGATTGTGCATCAGCTGACATGGTGGCC
NSP12	13442-16236	FP: GCACAGTGGCGGCCGCTCGAGGCCACCATGTCAGCTGATGCACAATCGTTT
		RP: GTCATCCTTGTAATCTCTAGACTGTAAGACTGTATGCGGTGTGTAC
NSP13	16237-18039	FP: GCACAGTGGCGGCCGCTCGAGGCCACCATGGCTGTTGGGGCTTGTGTTCT
		RP: GTCATCCTTGTAATCTCTAGATTGTAAAGTTGCCACATTCCTAC
NSP14	18040-19620	FP: GCACAGTGGCGGCCGCTCGAGGCCACCATGGCTGAAAATGTAACAGGACTCTTT
		RP: GTCATCCTTGTAATCTCTAGACTGAAGTCTTGTAAAAGTGTTCCAG
NSP15	19621-20658	FP: GCACAGTGGCGGCCGCTCGAGGCCACCATGAGTTTAGAAAATGTGGCTTTTAATG
		RP: GTCATCCTTGTAATCTCTAGATTGTAATTTTGGGTAAAATGTTTCT
NSP16	20659-21555	FP: GCACAGTGGCGGCCGCTCGAGGCCACCATGTCTAGTCAAGCGTGGCAACC
		RP: GTCATCCTTGTAATCTCTAGAGTTGTTAACAAGAACATCACTAGAAATA
**Structural proteins**		
S	21536-25384	FP: GCACAGTGGCGGCCGCTCGAGGCCACCATGTTCTTGTTAACAACTAAACGAAC
		RP: GTCATCCTTGTAATCTCTAGATGTGTAATGTAATTTGACTCCTTTGA
E	26245-26472	FP: GCACAGTGGCGGCCGCTCGAGGCCACCATGTACTCATTCGTTTCGGAAG
		RP: GTCATCCTTGTAATCTCTAGAGACCAGAAGATCAGGAACTCTAG
M	26523-27191	FP: GCACAGTGGCGGCCGCTCGAGGCCACCATGGCAGATTCCAACGGTAC
		RP: GTCATCCTTGTAATCTCTAGACTGTACAAGCAAAGCAATATTG
N	28274-29533	FP: GCACAGTGGCGGCCGCTCGAGGCCACCATGTCTGATAATGGACCC
		RP: GTCATCCTTGTAATCTCTAGAGGCCTGAGTTGAGTCAGCA
**Accessory proteins**		
ORF3a	25393-26220	FP: GCACAGTGGCGGCCGCTCGAGGCCACCATGGATTTGTTTATGAGAATC
		RP: GTCATCCTTGTAATCTCTAGACAAAGGCACGCTAGTAGTCG
ORF3b(opt)	25814-25882	FP: TCGAGGCCACCATGATGCCCACCATCTTCTTCGCCGGCATCCTGATCGTGACAACCA<br>TCGTGTACCTGACCATCGTGT
		RP: CTAGACACGATGGTCAGGTACACGATGGTTGTCACGATCAGGATGCCGGCGAAGAA<br>GATGGTGGGCATCATGGTGGCC
ORF6	27202-27387	FP: GCACAGTGGCGGCCGCTCGAGGCCACCATGTTTCATCTCGTTGACTTTCAG
		RP: GTCATCCTTGTAATCTCTAGAATCAATCTCCATTGGTTGCTC
ORF7a	27394-27759	FP: GCACAGTGGCGGCCGCTCGAGGCCACCATGAAAATTATTCTTTTCTTGGCAC
		RP: GTCATCCTTGTAATCTCTAGATTCTGTCTTTCTTTTGAGTGTGAAG
ORF7b	27756-27887	FP: GCACAGTGGCGGCCGCTCGAGGCCACCATGATTGAACTTTCATTAATTGACTTC
		RP: GTCATCCTTGTAATCTCTAGAGGCGTGACAAGTTTCATTATGA

ORF8	27894-28259	FP: GCACAGTGGCGGCCGCTCGAGGCCACCATGAAATTTCTTGTTTTCTTAGGAAT<br>RP: GTCATCCTTGTAATCTCTAGAGATGAAATCTAAAACAACACGAAC
ORF9a	28284-28577	FP: GCACAGTGGCGGCCGCTCGAGGCCACCATGGACCCCAAAATCAGCG<br>RP: GTCATCCTTGTAATCTCTAGATTTTACCGTCACCACCACGA
ORF9b	28734-28955	FP: GCACAGTGGCGGCCGCTCGAGGCCACCATGCTGCAATCGTGCTACAAC<br>RP: GTCATCCTTGTAATCTCTAGAATCTGTCAAGCAGCAGCAAAG
ORF10(opt)	29558-29674	FP: CTTGGTACCGAGCTCGGATCCGCCGCCACCATGGGCTACATCAACGTGTTCG<br>RP: GTCATCCTTGTAATCTCTAGAGGTCAGATTGAAGTTCACCACATCC

Table S1. ORFs of SARS-CoV-2 used in plasmid construction.

FR: Forward Primer; RP: Reverse Primer; nt: nucleartide; opt, codon optimized.
